# What can we do about Dr. Google? Using the electronic medical record (EMR) to prescribe reliable online patient education

**DOI:** 10.5195/jmla.2019.774

**Published:** 2019-10-01

**Authors:** Ruti Volk, Nabeel Obeid

**Affiliations:** Patient Education and Health Literacy Program Lead, Michigan Medicine, 1500 East Medical Center Drive, Ann Arbor MI 48109, rvolk@umich.edu; Assistant Professor, Department of Surgery, Michigan Medicine, 1500 East Medical Center Drive, Ann Arbor MI 48109, obeidn@med.umich.edu

## Abstract

**Objective:**

The project enabled clinicians to utilize the electronic medical record (EMR) to easily prescribe preapproved online patient education resources to their patients.

**Background:**

Physicians and other clinicians are concerned about the wide use of “Dr. Google” and the difficulties of responding to patients who demand unproven or unnecessary tests and therapies they found out about on the Internet.

**Setting/Participants/Resources:**

Participants were providers at a large health system using Epic EMR. The institution maintains a web-based database that links to print and electronic patient education materials that have been vetted by content experts.

**Methods:**

Clinicians worked with librarians to create web pages that link to the resources they recommend for their patients. Librarians collaborated with the information technology (IT) department to implement a solution that enables clinicians to quickly and easily send the uniform resource locator (URL) to the after visit summary (AVS) or as a message via the patient portal.

**Results:**

This solution has been implemented in more than 20 units across the institution. Analytics data demonstrate that the majority of patients in a surgery clinic visited recommended resources.

**Conclusion:**

This simple solution is effective in directing patients to reliable resources. It can be easily adapted by other institutions using an EMR system such as EPIC or Cerner.

Physicians have long been concerned about the wide use of “Dr. Google” and the difficulties of responding to patients who demand unproven or unnecessary tests and therapies they found out about online [1, 2]. Data from a 2013 national survey conducted by the Pew Research Center indicate that one in three American adults have gone online specifically to try to figure out what medical condition they or someone else might have [3]. Free access to vast amounts of medical information has many benefits, but if patients rely on inaccurate, outdated, or erroneous information to manage their health, it can lead to unnecessary anxiety and stress, medical mistakes, and bad outcomes. Several published studies recommend that physicians assume the responsibility of directing patients to quality online health information resources [2, 4]. A 2014 study concludes that “it is important to have a centralized ‘physician-certified’ online resource to which physicians could readily refer their patients without concern that they are receiving unreliable or misleading information” [5].

Michigan Medicine, the University of Michigan health system, is one of the largest hospitals in Michigan, a premier academic medical center, and one of the nation’s largest biomedical research communities. Providers at the institution use the Epic electronic medical record (EMR) system to prescribe reliable online health information resources to patients. This solution relies on a web-based database, the Michigan Medicine Patient Education Clearinghouse, that includes patient education materials that have been created or approved by Michigan Medicine’s experts.

The clearinghouse, implemented in 2010, is a bibliographic database designed by a librarian and built on a Drupal platform. Each record in the database includes more than thirty-five fields that enable cataloging with many descriptors such as material type, format, language, population, keywords, and subject headings from the Consumer Health Vocabulary (CHV), which is part of the Unified Medical Language System (UMLS).

Created initially for clinicians, the clearinghouse includes a second, dedicated interface designed specifically for patients and caregivers. This Care Guides from Your Clinician interface, implemented in 2012, uses plain language and navigation that is intuitive for patients rather than clinicians. It is available for free without registration or login. The care guides interface includes sub-pages created especially for patients seen in specific clinics. To create clinic-specific collections, clinicians submit relevant topics to librarians, who suggest relevant existing materials. If a gap is identified, librarians help locate or create new materials that are added to the database. An example of the Care Guides sub-page is one created for patients who are seen at the Minimally Invasive Surgery Clinic.

To direct patients to the Care Guides site, librarians created special flyers called “Education Rx” with the uniform resource locator (URL) to the clinic-specific sub-page and a short description of what the user would find on the sub-page. These print materials were handed to patients or hung in exam rooms. In 2018, at the request of clinicians, librarians started to upload the Education Rx flyers as patient instruction documents into the Epic EMR. This process involves librarians preparing and uploading documents to a cloud storage system and information technology (IT) staff importing them into Epic. The Patient Instructions feature of Epic enables providers to send the flyers, which have been reformatted into “Take-Home Instructions for the Patient,” to print with the after visit summary (AVS) or as a message via the patient portal. Figure 1 shows an AVS with “Take-Home Instructions for the Patient” that would be printed out for patients at the conclusion of each visit.

**Figure 1:**
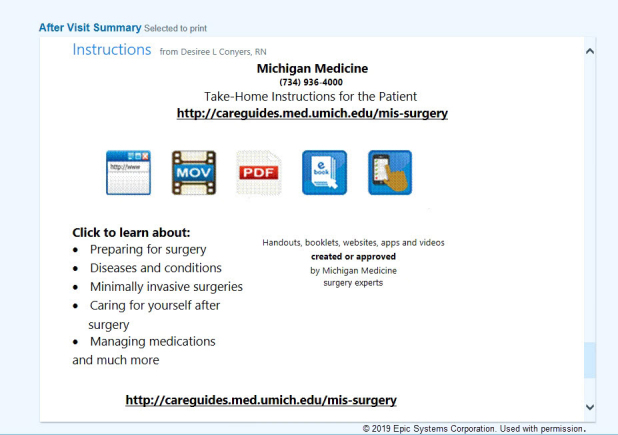
Patient after visit summary

Over time, this service has grown, and Education Rx information included with the AVSs or in the patient portal has been implemented in more than 20 units across the institution. Table 1 summarizes patient data collected during the first year of implementation in the Minimally Invasive Surgery Clinic in Chelsea, Michigan, and web analytics data for the Minimally Invasive Surgery Care Guides page between May 1, 2018, and April 30, 2019. During this time period, 278 unique patients were seen at the clinic, and the Education Rx was sent to print with the AVS 706 times. The URL was accessed by 252 new visitors and 122 returning visitors. The data demonstrated that patients received the Education Rx information multiple times over several visits and that the Education Rx information with the AVS and patient education page was actively used. Because the number of visitors to the page was similar to the number of patients seen at the clinic, this provides some confidence that the data accurately reflects usage during this period.

**Table 1:** Patient data collected during the first year of implementation in the Minimally Invasive Surgery Clinic

Data	n
Number of unique patients seen at the clinic	278
Number of times Education Rx was sent to print with the AVS*	706*
Page views	598
New visitors to the web page	252
Returning visitors to the web page	122

* Patients have multiple visits at various locations.

This solution of providing patient education information with the AVSs or in the patient portal has proved to be an effective strategy in directing patients to reliable resources at our hospital. This model can be adapted by other institutions using an EMR system such as Epic or Cerner. Based on active usage and clinicians’ satisfaction with this project at the Minimally Invasive Surgery Clinic, plans are underway to expand this solution into other ambulatory care units at Michigan Medicine.

**Ruti Volk, MSI, AHIP**, rvolk@umich.edu, https://orcid.org/0000-0002-8729-7614, Patient Education and Health Literacy Program Lead, Michigan Medicine, 1500 East Medical Center Drive, Ann Arbor MI 48109

**Nabeel Obeid, MD**, obeidn@med.umich.edu, https://orcid.org/0000-0002-5509-6527, Assistant Professor, Department of Surgery, Michigan Medicine, 1500 East Medical Center Drive, Ann Arbor MI 48109
